# Guardians of immunity: the role of tumour-draining lymph node in cancer immunity

**DOI:** 10.3389/fcell.2026.1816042

**Published:** 2026-05-22

**Authors:** Darellynn Oo, Isha Sharma, Jingna Xue, Shan Liao

**Affiliations:** Department of Microbiology, Immunology and Infectious diseases, Snyder Institute for Chronic Diseases, Cumming School of Medicine, University of Calgary, Calgary, AB, Canada

**Keywords:** B cell, immune check inhibitor, immunotherapy, tertiary lymph structures, tumor draining lymph node (TDLN)

## Abstract

The success of immunotherapies in cancer has changed the landscape of cancer research. The tumour-draining lymph node (TDLN) is emerging as an organ specific target to enhance the efficacy and minimize the toxicity of immunotherapies; this approach is based on the TDLN being the first lymph node to encounter metastatic tumour cells and various tumour-derived factors. Both anti-tumour and immunosuppressive T cells can originate from the TDLNs. Accumulating evidence has shown that immunosuppressive T cells are activated in the TDLN before migrating to the tumour to perform their functions. This suggests that targeting the TDLN could be crucial for intercepting immunosuppressive T cells and enhancing immunotherapy. Notably, regional delivery of ICI drugs to the TDLN has been shown to achieve similar outcomes to systemic delivery but with reduced toxicity to the host. T cell immunity is well-known to be regulated by dendritic cells (DCs) in cancer. However, B cells, which form a significant population of immune cells in the TDLNs, have emerging roles in this regulation. This review seeks to elucidate the mechanisms of cancer immunity within the TDLN and to explore the potential role of B cells in immunotherapies.

## 1 Introduction

Lymphatic metastasis is a prominent feature of solid cancers, including breast, melanoma, head and neck, lung, and colorectal cancer. Clinically, metastasis to the sentinel lymph nodes, also called TDLNs, correlates with a poor patient prognosis and is an essential marker for staging and decisions on therapy regimens. TDLNs may function as an intermediary rest area before cancer cells spread to distal organs, as evidenced by a few studies showing metastatic cancer cells can invade the blood vessels in the TDLNs and subsequently spread to the lung and bone marrow (Brown et al., 2018; Pereira et al., 2018). Therefore, a standard procedure for cancer surgery includes tumour resection and lymphadenectomy. Lymphadenectomy is a surgical procedure to resect lymph nodes (LNs) aimed at reducing the risk of tumour recurrence. Nevertheless, the clinical benefits of lymphadenectomy remain controversial (Connolly et al., 2021; Damo et al., 2021; Li et al., 2022). Emerging evidence suggests that the TDLN plays a vital role in cancer immunoregulation, including educating tumour-specific T cells (Bikah et al., 1996; Glade Bender et al., 2008). Removal of the TDLN also removes locally “educated” immune cells, and how this impacts anti-tumour responses or immunotherapy remains unclear. Herein, we will discuss the role of the TDLN in cancer immunoregulation and cancer immunotherapy, as well as how it can serve as a promising juncture for therapeutic intervention in the first part of the introduction.

While dendritic cells (DCs) are important antigen-presenting cells (APCs) that prime antigen-specific T cells in the TDLN, other cell types may also regulate T cells in TDLNs. B cells have been shown to promote immunosuppressive T cells in the TDLN (Ganti et al., 2015). Despite this, the role of B cells in cancer immunoregulation remains poorly understood, largely because the number of tumour-infiltrating B cells is typically small and often overlooked by mainstream research. In contrast to tumour-infiltrating B cells, B cells constitute 40%–50% of the immune cells in the TDLN and comprise diverse populations that can exert both anti-tumour and pro-tumour effects. When B cells are activated, they can rapidly differentiate into IgM-expressing plasmablasts, plasma cells and memory B cells through a germinal centre (GC)-independent manner. It takes an additional few days for B cells to undergo GC-dependent maturation and class switch to differentiate into high-affinity antibody-producing IgG and IgA plasma cells and memory B cells. Several tissue-resident B cells, including B-1 cells and IgM^+^ plasma cells, reside in lymphoid organs and body cavities and play immunoregulatory roles. These cells express immunosuppressive cytokines such as IL-10, IL-35, and TGF-β. Unfortunately, there is no consensus on surface markers defining these B cell subsets. Thus, identifying B cell subsets, especially those with immunoregulatory functions, is crucial for targeting specific B cell populations to enhance anti-tumour immunity. In the second part of this introduction, we will discuss current knowledge regarding B cells, particularly regulatory B cells (B_Regs_), in cancer immunoregulation and immunotherapy, as well as their potential as therapeutic targets within the TDLN.

## 2 Lymph node (LN): immune response and peripheral tolerance

**FIGURE 1:**
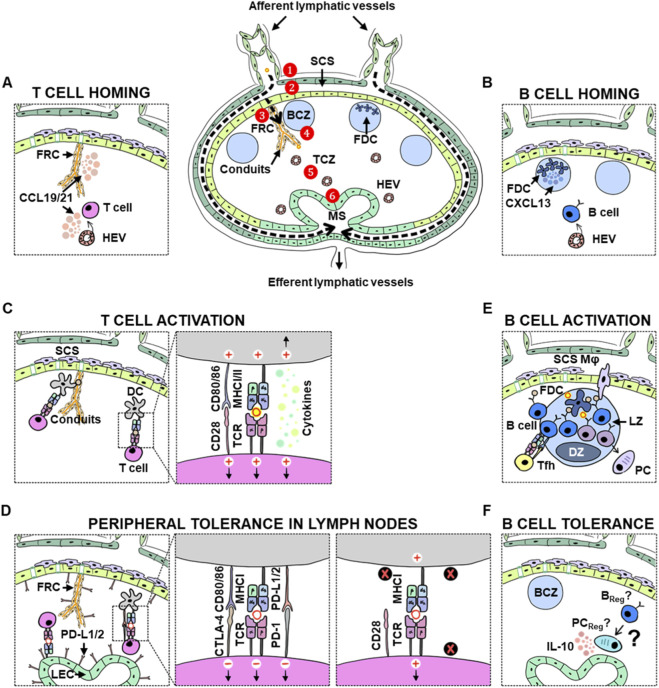
Lymph node (LN) structure and function: Lymph node microarchitecture is complex and can be broken down into the following: 1) capsule, the outermost fibrous layer; 2) subcapsular sinus (SCS), the space between the capsule and parenchyma, which collects lymph from the afferent vessels for filtration; 3) fibroblastic reticular cells (FRC) and conduit network, a dense extracellular matrix (ECM) that filters lymph; 4) cortex, which houses B cells; 5) paracortex, which houses T cells; 6) high endothelial venules (HEV), specialised post-capillary vessels that facilitate immune cell migration to and from the bloodstream; 7) medullary sinus (MS), which mainly comprise of plasma cells and macrophages, and is the site where fluid can exit from the efferent vessels. **(A)** T cell homing to the LNs. **(B)** B cell homing to the LNs. **(C)** Multiple signals facilitate T cell activation in the LNs. **(D)** Immunosuppression and peripheral tolerance mechanisms in the LNs are coordinated by dendritic cells (DC), lymphatic endothelial cells (LEC) and FRCs. **(E)** B cell activation and germinal centre reaction in the LNs require orchestration of signals from follicular dendritic cells (FDC) and follicular T-helper cells (Tfh). **(F)** B cells may also possess immunoregulatory functions by secreting immunosuppressive cytokines, but the mechanism remains poorly understood.

The lymphatic system functions as the body’s sewage system, removing extra fluid and tissue waste from the tissues. Tissue wastes, such as metabolic by-products and cellular debris, are absorbed by lymphatic vessels with lymph, then drained to the LNs via afferent lymph vessels. Lymph-borne materials are surveyed by cells in the LNs for pathogens and other danger signals for immune protection. The filtered lymph and immune cells that have completed immunosurveillance exit LN via efferent lymphatic vessels and return to circulation (Figure 1). In the LN, lymphatic endothelial cells (LECs) line the subcapsular and medullary regions of the LN to form subcapsular sinus (SCS) and medullary sinuses (MS) to support lymph and cells to enter or egress the LN via lymphatic vessels (Figure 1). LN sinus-associated immune cells are primarily macrophages, some dendritic cells (DCs), a small proportion of natural killer (NK) cells, NKT cells, γδT cells and other innate immune cells. The majority of immune cells (>90%) in the LN are T and B cells, organised into T and B cell zones (Figure 1). Naïve T and B cells enter the LN via specialised vascular structures called high endothelial venules (HEVs) and patrol the LN for potential pathogens. Blood vessel endothelial cells (BECs) lining the HEVs are specialised in helping immune cells home to the LN by expressing selectins, integrins and chemokines. HEVs and fibroblastic reticular cells (FRCs) in the T cell zone and the interfollicular zone, the area between B cell follicles, secrete chemokines CCL19/CCL21 to guide DC and T cell trafficking. Additionally, FRCs wrap around conduits, a labyrinthine network of extracellular matrix that drains lymph deep into the LN to facilitate materials in lymph reaching DCs and T cells (Figure 1A). In the B cell zone, follicular dendritic cells (FDCs) express chemokine CXCL13 to attract CXCR5-expressing B cells, forming B cell follicles (Drayton et al., 2006; Montorsi et al., 2022) (Figure 1B).

Upon antigen encounter, the lymph carries free-form antigens from the peripheral tissue to the LN within a few minutes. Antigen-presenting cells (APCs) that reside in the LN, particularly DCs located near the SCS and conduits, can acquire lymph-borne antigens to initiate the first wave of T cell activation (Figure 1C). A few hours later, tissue-migrating DCs that have acquired antigens upregulate chemokine receptors, enabling them to enter lymphatic vessels and travel to the LNs to prime antigen-specific T cells (Figure 1C). Canonically, DCs present endogenous antigens via MHC Class I to CD8 T cells and exogenous antigens on MHC Class II to CD4 T cells; however, DCs also possess the ability to cross-present antigens by processing exogenous antigens and presenting them on MHC Class I to CD8 T cells. To fully activate T cells, DCs upregulate co-stimulatory molecules CD80/CD86 to engage CD28 on T cells and secrete cytokines to promote T cell proliferation and differentiation (Figure 1C). After antigen clearance, most proliferated T cells must be eliminated to avoid detrimental effects such as generating autoreactive T cells. Hence, negative feedback mechanisms constraining T cell activation, such as immune checkpoint molecules, are crucial regulators in T cell immunity. Activated T cells may upregulate CTLA-4, which binds to CD80/CD86 on DCs with higher affinity, outcompeting CD28 (Figure 1D). CTLA-4 then delivers downstream inhibitory signals to dampen T cell activation. Inhibitory molecules like CTLA-4 thereby function as immune checkpoints or “brakes” to ensure immune responses are contained at specific locations and for specific periods. Another well-studied immune checkpoint molecule is PD-1, which is linked to T cell exhaustion. T cell exhaustion is a state of T cell dysfunction whereby T cells lose their effector capacity (Figure 1D) (Pauken and Wherry, 2015; Blank et al., 2019). The PD-1/PD-L1 axis has since become a hallmark of T cell exhaustion and has been well-documented in chronic infections and cancers (Pardoll, 2012; Zou et al., 2016).

B cells, on the other hand, are positioned close to the SCS, where lymph enters the LN. This strategic positioning allows them to efficiently capture antigens or immune complexes from incoming lymph. At early time points, upon antigen encounter, B cells can differentiate into low-affinity IgM plasmablasts or plasma cells to provide rapid antibody protection. FDCs, the stromal cells responsible for supporting B cell follicles, are located close to the SCS and are poised for the easy acquisition of antigens in the lymph. Additionally, macrophages in the SCS can acquire antigens and deposit them on FDCs (Figure 1E). Antigen-loaded FDCs play an essential role in germinal centres (GCs). With the support of follicular T helper cells (Tfh) and FDCs, activated B cells form GCs and cycle back and forth within the light and dark zones of the GCs in a process called affinity maturation. In the dark zone (DZ), B cells undergo active proliferation and somatic hypermutation. Antigen presentation by FDCs is a crucial step in the light zone (LZ), which helps select the B cells that express high-affinity antibodies to the cognate antigens. High-affinity B cells can undergo isotype switching to differentiate into memory B cells and antibody-secreting plasma cells (Figure 1E). The movement of B cells between the light and dark zones is guided by the expression of the chemokine receptor CXCR4 and its chemokine, CXCL12. Some B cells can bypass T cell help and undergo GC-independent responses. GC-independent responses are commonly induced by lipopolysaccharides (LPS) or CpG stimulation, which lead to IgM plasma cells and low-affinity antibody production.

Another critical function of LNs is to manage peripheral tolerance and prevent autoimmunity. During T cell development in the thymus, T cells undergo central tolerance, during which autoreactive T cells lose their function and undergo anergy or clonal deletion. Anergy is a state of inactivation in which immune cells can no longer respond to antigens. Clonal deletion occurs when autoreactive cells die via apoptosis. Some autoreactive T cells are reprogrammed into regulatory T cells (T_Regs_) in the thymus, which can help modulate T cells. However, some self-reactive T cells may still escape into the periphery. As a fail-safe, LNs act as another layer of protection by maintaining peripheral tolerance. In the LNs, peripheral tolerance is primarily regulated by DCs. DCs that present self-antigens but lack co-stimulatory molecules or express tolerogenic molecules, help to induce anergy or clonal deletion of autoreactive T cells (Figure 1D) (Steinman et al., 2003). Additionally, lymph node stromal cells (LNSCs) also play a role in peripheral tolerance (Figure 1D) (Collier et al., 2008; Mueller, 2009; Fletcher and Heng, 2016)FRCs and LECs in the LN can express and present peripheral tissue antigens (PTAs) via MHC Class I and express PD-L1 to induce anergy and clonal depletion of autoreactive T cells (Lee et al., 2006; Cohen et al., 2010; Fletcher et al., 2010). LECs and FRCs also promote peripheral tolerance by presenting through MHC Class II to CD4 T cells or inducing T_Regs_ (Baptista et al., 2014; Nadafi et al., 2020). Therefore, in addition to their conventional roles in generating an immune response to foreign antigens, LNs play a vital immunoregulatory role. Lymph nodes may harbour a small subset of B_Regs_, but compared to immunoregulatory T cells, their identity and roles remain poorly understood (Figure 1E) (Byrne and Halliday, 2005; Natarajan et al., 2012; Wilson et al., 2019).

## 3 Cancer immunity and the TDLN

**FIGURE 2:**
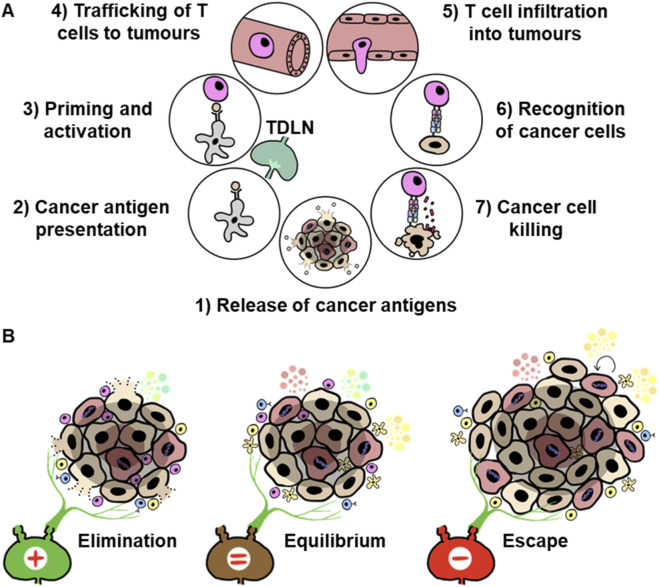
Cancer-Immunity cycle and immunoediting: **(A)** Adapted from Chen and Mellman (2013), the cancer immunity cycle is a seven-step process depicting how host immunity detects and eradicates cancer cells. The TDLN plays a vital role as the site of priming and educating tumour-specific T cells. **(B)** Adapted from Dunn et al. (2002), the 3 E’s of cancer, elimination, equilibrium, and escape, show how host immunity interacts with cancer cells and how these interactions can indirectly lead to the selection of immune-evasive tumour variants. Specifically, the tumour microenvironment (TME) can impact the TDLN, shifting it from responding to the tumour to becoming tumour-permissive.

Cancer cells constantly divide, increasing the propensity to develop genetic mutations. An accumulation of these mutations leads to increased tumour heterogeneity and the formation of new variants. These mutations may become cancer antigens to activate host immunity. Depending on the origin, cancer antigens encompass a variety of antigens, including virus-specific antigens, overexpressed antigens, fusion peptides and spatiotemporally dysregulated antigens (Schumacher and Schreiber, 2015; Schumacher et al., 2019). Even though some tumour-associated antigens are more immunogenic, whilst others are less likely to be detected by the immune system, TDLNs can respond to various types of tumour-associated antigens (Munn and Mellor, 2006; du Bois et al., 2021). Chen and Mellman elegantly simplified cancer-host immune responses into the Cancer-Immunity cycle (Chen and Mellman, 2013). Firstly, DCs in the primary tumour recognise and acquire cancer antigens. Activated DCs then traffic to the TDLN to prime and activate T cells. Subsequently, these cancer-specific T cells can home to tumours to elicit anti-tumour responses (Figure 2A).

One of the crucial steps of cancer progression is to escape the Cancer-Immunity cycle. In 2022, Dr Schreiber coined the term “immunoediting”, famously known as the 3 E’s: elimination, equilibrium, and escape, to address different stages of host immunity against cancers (Dunn et al., 2002). In elimination, the immune system can recognise and mount an effective immune response against the cancer cells to limit their growth. In some cases, the cancer cells are eliminated if the immune cells are successful. However, due to the genomic instability of cancer cells, they may acquire genetic or phenotypic alterations that enable them to survive these attacks. Subsequently, if the rates of killing the cancer cells are similar to the cancer cell’s ability to replicate, or if the cancer cells enter a state of dormancy undetectable by immune cells, it results in equilibrium. Once the cancer cells surpass the host immune defences, it culminates in the last step, escape. As the TDLNs encounter metastatic tumour cells, tumour-associated antigens, and various immunoregulatory factors from the tumour, TDLNs can react to the tumour by activating antigen-specific T cells. However, when the tumour microenvironment gradually changes to favour cancer progression, so does the TDLN. Additionally, since cancer cells are inherently self and can exploit tolerogenic mechanisms, the TDLN may become immunotolerant towards the cancer (Figure 2B). Hence, understanding how the TDLN can switch from anti-tumour responses to immune tolerance to tumours will allow scientists to design novel strategies to augment cancer immunotherapy.

### 3.1 Cancer immunity in the TDLN: anti-tumour immunity

**FIGURE 3:**
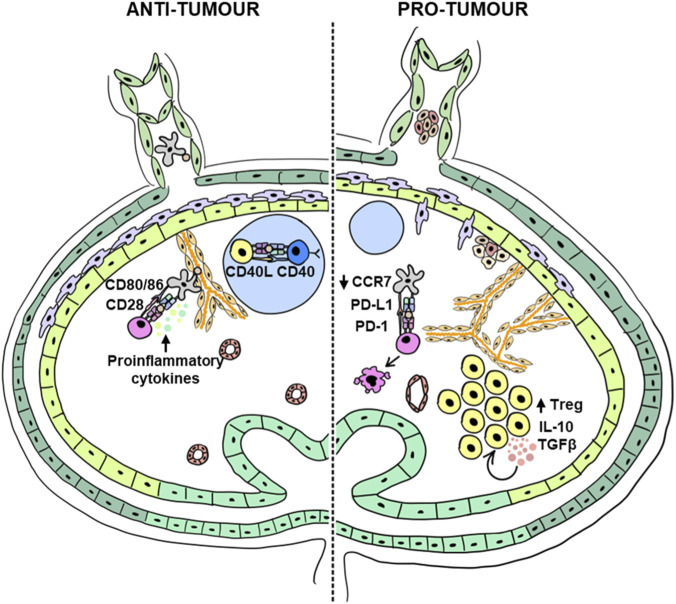
T cell cancer immunity in the TDLN: Left (Anti-tumour immunity): The TDLN, a site of dynamic immune activity, enables optimal interactions between immune cells such as DCs and cytotoxic T cells, leading to potent anti-tumour responses against the tumour. Similarly, T-helper cells play a crucial role by providing necessary signals to assist other immune cell populations, as seen in the interactions between Tfh and B cells. Right (Pro-tumour immunity): However, the TDLN can also shift to an immunosuppressive or pro-tumoural response, a key factor in cancer metastasis. Immune cells in the TDLN undergo significant changes, becoming tolerogenic, such as changes in co-stimulatory molecules and induction of immune checkpoint molecules to promote the expansion of T_Regs_ and induce T cell exhaustion, which dampens cytotoxic T cell responses. This dual nature of the TDLN underscores its complexity and importance in cancer immunity and metastasis.

To successfully eradicate tumour cells, the Cancer-Immunity cycle must be efficiently initiated, maintained and propagated (Chen et al., 2013). Priming phase kick-off with the release of tumour associated antigens (TAAs) and DAMPs such as ATP, HMGB1 or calreticulin. DAMPs act through TLRs to mature and activate DCs for migration (Maiorino et al., 2021). DCs are widely accepted as the initiator of anti-tumour immunity (Figure 3) (Marciscano and Anandasabapathy, 2021; Mellman, 2013). In the TME, DCs come in contact with tumour derived antigens. Mature DCs migrate to TDLN to cross-present TAA to naïve T cells. T cell activates when the T cell receptor aligns with the antigen and obtains positive co-stimulatory signals (e.g., CD80/86 response to CD28). DCs that tend to activate CD8 T cells are classified as type I conventional DCs (cDC1), and those that activate CD4 T cells are type II conventional DCs (cDC2). The classification can be better defined based on their surface markers, cytokine production, and transcription factors (Collin and Bigley, 2018; Guermonprez et al., 2019). In melanoma patients, tumour antigen-loaded DCs express CCR7 and can home to TDLN, specifically the T cell zone to help T cells survival, proliferate and function. cDC1 maintain standard functioning of CD8 T cells through IL-12 and IL-15 (Oncology, 2003; Böttcher and Reis e Sousa, 2018). Similarly, in a melanoma mouse model and patients, cDC1s were found to migrate to the TDLN in a CCR7-dependent manner guided by CCL19/CCL21 gradients (Roberts et al., 2016). Without CCR7, these DCs could not migrate to TDLN, and the anti-tumour effects are abrogated. Lei et al., 2023, showed that cDC1 requires CD4^+^ T cells to help cross-present antigens or prime CD8^+^ T cells (Lei et al., 2023; Meiser et al., 2023).

Effector phase of anti-tumour immunity involves trafficking and infiltration of CD8+T cell towards CXCL9 and CXCL 10 gradients. Cytotoxic T cells recognize antigens on tumour surface and induce apoptosis of tumour cell (Chang and Beatty, 2020). In return the dead tumour cells release more TAA which maintains the immune response as long as antigen is present. This response is suppressed by T_Regs_, MDSCs M2-type macrophages and kept in check by PD-1 and CTLA-4 (Chang and Beatty, 2020).

### 3.2 Cancer immunity in the TDLN: immunosuppression

Many studies have shown that T cell responses are repressed in the TDLNs, which may exist long before metastasis occurs (Mansfield et al., 2011). T_Regs_, known for their potent immunosuppressive capabilities, have been consistently associated with poorer patient prognoses in various cancer types, including breast cancer, melanoma, and ovarian cancer (Curiel et al., 2004; Viguier et al., 2004; Nakamura et al., 2009; Li et al., 2016). These cells are capable of secreting IL-10, IL-35 and TGF-β, which promote T_Reg_ generation and inhibit cytotoxic CD8 T cell, Th1, and Th2 differentiation (Dercamp et al., 2005; Vignali et al., 2008). Since T_Regs_ can secrete a plethora of immunosuppressive cytokines to induce more T_Regs_, this creates a positive feedback loop for their expansion. Furthermore, these immunosuppressive cytokines can inhibit cytotoxic CD8 T cells, aiding cancer immune evasion. Plasmacytoid DCs (pDCs) have also been implicated in inducing T_Reg_ through various mechanisms (Sharma et al., 2007; Gai et al., 2013). In addition to T_Regs_, cancer cells, stromal cells and other immune cells can also produce immunosuppressive cytokines for immune evasion (Turley et al., 2015; Mao et al., 2021).

Several studies have also explored the function of T_Regs_ in the TDLN. For instance, in colon cancer, it was found that TGF-β from myeloid-derived suppressor cells (MDSCs) promoted T_Reg_ expansion in the TDLN (Ghiringhelli et al., 2005). In node-positive breast cancer patients, the TDLNs exhibited less IFNγ expression but contained more T_Reg_ and Th2 cells (Faghih et al., 2014). Núñez et al. also reported that the TDLN of breast cancer patients accumulate T_Regs_. Moreover, T_Regs_ isolated from the TDLN retain their immunosuppressive capabilities *ex vivo* and express a wide array of checkpoint molecules (Núñez et al., 2020). Additionally, the authors found that TDLN T_Regs_ were transcriptionally like Type 1 T regulatory cells (Tr1) and T follicular regulatory cells (Tfr), which may promote more tolerance and inhibit humoral immunity within the TDLN. Tfrs have also been documented in TDLNs in breast cancer patients (Faghih et al., 2014). This implies that not only can T_Regs_ impair T cell response in the TDLN, but B cell responses may also be affected. Therefore, T_Regs_ play a prominent role in cancer-mediated immunosuppression. Since systemically eliminating T_Regs_ can result in severe autoimmunity, locally targeting T_Regs_ in the TDLN may be a feasible alternative.

Maintaining DC-T cell interaction is necessary to attain adequate co-stimulation. While mature DCs can drive anti-tumour immunity by presenting tumour antigens to T cells, cancer-associated DCs are often immature DCs lacking CCR7 or co-stimulatory molecules. In melanoma, DCs may lack CCR7, thus impairing their ability to get to the TDLN and effectively mount an immune response (Oncology, 2003). Melanoma-derived extracellular vesicles can also impair DC maturation and reduce Th1 response (Maus et al., 2017). T cell activation is tightly regulated, and insufficient contact time can prevent T cells from becoming fully activated. One study showed that CTLA-4 impacts T cell motility by reducing contact time and interaction between DCs and T cells (Schneider et al., 2006).

As previously mentioned, the PD-1/PD-L1 axis has become a hallmark in cancer for T cell exhaustion. Cancer cells can upregulate immune checkpoint molecules for contact-dependent immunosuppression. PD-1 must engage its ligand PD-L1 to induce T cell dysfunction and exhaustion. PD-1^+^ T cells are frequently identified in the primary tumour. Simultaneously, cancer cells, tumour-associated macrophages (TAMs), DCs and stromal cells often upregulate PD-L1, thereby causing T cell exhaustion within the tumours. Recently, it has been shown that PD-1^+^ CD8 T cells are first primed in the TDLN before migrating to the tumour (Dammeijer et al., 2020). The same study also demonstrated that cDC2s upregulate PD-L1 and potentially interact with and suppress CD8 T cells.

Moreover, LECs in the LNs play a pivotal role in maintaining peripheral tolerance by presenting self-peptides and expressing PD-L1 (Figure 1D). A recent study by Cousin et al. demonstrated that conditionally depleting PD-L1 in LECs led to the expansion of tumour-specific CD8^+^ T cells in TDLNs, suggesting that LECs in the TDLN may present tumour-associated antigens and suppress anti-tumour T cell responses (Cousin et al., 2021). This finding underscores how cancer can exploit tolerogenic mechanisms in the LNs to facilitate its escape. These studies collectively support the notion that the TDLN may be more amenable to reactivation with immunotherapy than the primary tumour (Chow et al., 2022).

## 4 The TDLN as an immunotherapeutic target

Inhibitors of the PD-1/PD-L1 axis or CTLA-4 are the most commonly used ICI therapies. More checkpoint molecules have now been added to the list of emerging immunotherapeutic targets for cancer, such as LAG-3 and TIM-3. LAG-3 has been implicated in fine-tuning T cell responses (Hu et al., 2019). LAG-3-deficient CD8^+^ T cells had more effector function as measured by IFNγ and Granzyme B expression (Woo et al., 2012; Andrews et al., 2024). In breast cancer, TIM-3 upregulation in the TDLN was found on CD8^+^ T cells, CD4^+^ T cells and T_Regs_. TIM-3 also correlated with patients with higher-grade tumours or more LN involvement (Shariati et al., 2020). The discovery of novel checkpoint molecules allows researchers to target multiple checkpoint molecules to augment therapeutic effects.

While most research efforts concentrate on targeting tumour-infiltrating T cells through systemic delivery of ICIs, T cells in the tumour may be overwhelmed by the immunosuppressive microenvironment. ICI therapy may not fully restore T cell function due to low drug penetration within tumours, which may contribute to poor responsiveness in some patients (Minchinton and Tannock, 2006). Current efforts include examining different approaches to improve ICIs. The most common strategy to improve responsiveness in patients is combination therapy. Despite achieving relative success, combination therapy comes with adverse effects, such as increased toxicity. Hence, other approaches are required to help mitigate these side effects. Since the TDLN play an integral role in modulating immune responses, recent studies have focused on targeting the TDLN. This is supported by studies showing the induction and accumulation of T_Regs_ and PD-1^+^ T cells in the TDLN, which hint at the possibility of targeting immunosuppressive T cells locally in the TDLN (Fife and Bluestone, 2008; Kanda et al., 2021; van Pul et al., 2021; Yost et al., 2021; Piersiala et al., 2022). PD-1^+^ T cells have been found in the TDLN of mice in several cancers, such as mesothelioma, melanoma, and breast and colon cancer (Fransen et al., 2018; Dammeijer et al., 2020; Francis et al., 2020). Notably, some of these studies have concomitantly shown that local administration of ICI to the TDLN regressed tumours in mice models (Fransen et al., 2018; Dammeijer et al., 2020; Francis et al., 2020). Dammeijer et al. showed that in a mesothelioma mouse model, intrapleural injection of αPD-1 antibody allows delivery to the TDLN, which was as efficacious as systemic delivery (Dammeijer et al., 2020). Likewise, Francis et al. also found that locally delivering ICIs to the TDLN was beneficial and significantly limited the spread of the drug, consequently limiting toxicity in mice (Francis et al., 2020). Moreover, another study demonstrated that surgical resection of the TDLN reduced the efficacy of ICIs, underlining the significance of the TDLN. In the same study, using FTY720 to abrogate immune cell egress from the TDLN similarly reduced ICI efficacy. Hence, this supports the idea that T cell priming occurs in the TDLN and is essential for the outcome of ICI therapy (Fransen et al., 2018). These pieces of evidence demonstrate the importance of the TDLN in ICI therapy.

As depicted in the Cancer-Immunity cycle, priming of T cells within the TDLN precedes T cell homing to the tumour. Since CTLA-4 mainly functions through the inhibition of the co-stimulatory molecule CD28, it is thought that CTLA-4-mediated inhibition predominantly occurs within the TDLN (Fife and Bluestone, 2008; Buchbinder and Desai, 2016). A recent study showed that in early-stage melanoma patients, administration of αCTLA-4 to the excised melanoma site resulted in DC and T cell activation in the TDLN, as well as systemic effects such as reduced T_Regs_ and MDSCs in the PBMC (Notohardjo et al., 2020; van Pul et al., 2022). These results show that the efficacy of αCTLA-4 therapy can be achieved independently of the primary tumour. Apart from reactivating existing tumour-specific T cells, single-cell RNA sequencing data showed that ICIs might induce new tumour-specific T cell clones to aid anti-tumour immunity (Yost et al., 2021; Liu et al., 2022). Accumulating evidence suggests TDLNs serve as a more effective site for ICI therapies. Therefore, a better understanding of the dynamic immune responses within TDLNs will help inform decisions to optimise ICI delivery.

## 5 Bilateral roles of B cells in cancer immunity

**FIGURE 4:**
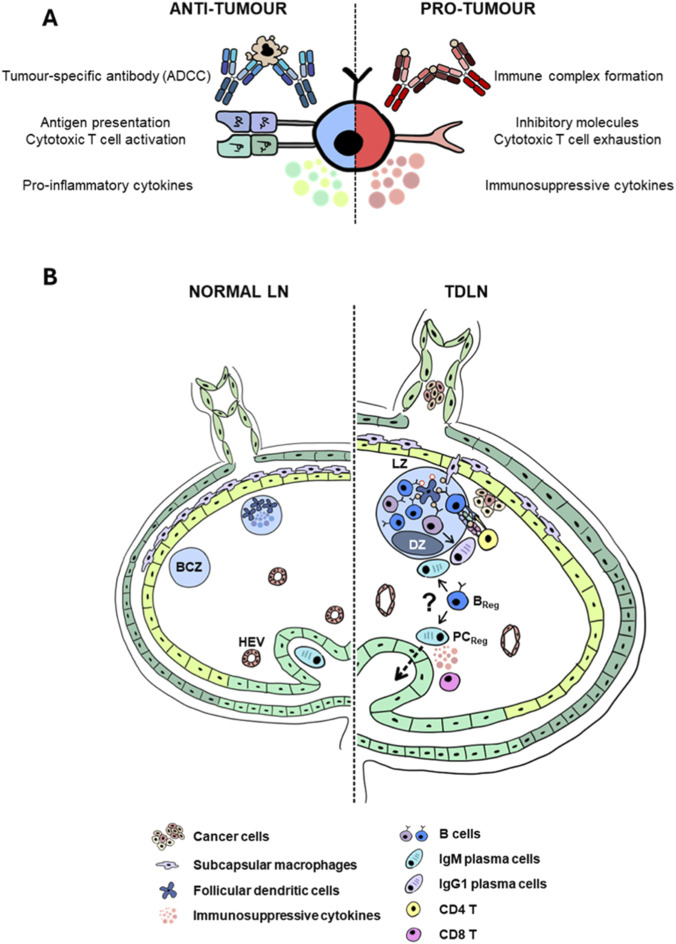
B cells in cancer immunity. **(A)** B cells carry out three main functions: 1) Antibody production, 2) Antigen presentation, 3) Secreting cytokines. Left: B cells aid anti-tumour immunity by producing tumour-specific antibodies, activating cytotoxic T cells and secreting pro-inflammatory cytokines that can assist T-helper polarisation into pro-inflammatory phenotypes. Right: Conversely, antibodies secreted by B cells can form immune complexes that lead to chronic inflammation, exacerbating cancer growth. B cells can express inhibitory molecules that modulate T cell function, causing T cell dysfunction or exhaustion. B_Regs_ can also secrete immunosuppressive cytokines such as IL-10, IL-35 and TGF-β. **(B)** B cell in the TDLN. Left: B cells in the LN during homeostasis. Right: B cell functions in the TDLN are diverse and remain unclear. B cells can undergo germinal centre reactions to produce plasma cells or be activated extrafollicularly to generate extrafollicular plasma cells. The TDLN may harbour both anti-tumoural and pro-tumoural repertoires of B and plasma cells. The signals underpinning how B_Regs_ and PC_Regs_ are produced are unclear. B cell-derived lymphotoxin signalling is a critical regulator of the lymph node microenvironment, including maintaining the subcapsular macrophages and follicular dendritic cells; thus, it may also be important in modulating the TDLN microenvironment.

Although B cells represent a large proportion of the immune cell repertoire in the TDLN, the role of B cells in cancer is understudied compared to T cells. Like T cells, B cells are heterogeneous and play both anti-tumour and pro-tumour roles (Figure 4A). B cells often account for a relatively small population of tumour-infiltrating immune cells and, thus, are easily neglected by mainstream cancer research. Furthermore, the lack of sufficient distinct markers to identify B cell subpopulations makes determining their roles in cancer immunity challenging. To date, the function of B cells in cancer remains highly contentious. Whether B cells are candidate targets for improving cancer immunotherapy remains to be determined.

B cells are mainly responsible for humoral immunity, which is to produce antibodies (Figure 4A). In the TDLNs, B cells can acquire tumour-associated antigens, become activated and seek help from T-helper cells. Activated T-helper cells express CD40L, which binds to CD40 on B cells (Klein and Dalla-Favera, 2008; Neumann et al., 2014). CD40/CD40L engagement is one of the primary signals required for B cells to form GCs, interact with FDCs, undergo selection and maturation, and differentiate into high-affinity memory B cells or plasma cells (Figure 4B). Although it is widely accepted that IgG antibodies aid in eradicating cancer cells, it is now known that pathogenic IgG antibodies may support metastasis (Gu et al., 2019). Antibodies may also cause complement activation, exacerbating chronic inflammation in the primary tumour and promoting tumour growth (Vadrevu et al., 2014; Kwak et al., 2018).

B cells also function as APCs and can directly interact with T cells (Figures 4A,B). In lung adenocarcinoma (LUAD), antigen-specific B cells in the TDLN cooperated with follicular T-helper cells (Tfh) to induce IL-21 production. Subsequently, IL-21 acted on CD8^+^ T cells to promote anti-tumour response. The authors also showed that anti-tumour effects can be abolished by knocking out B cells or disrupting the interaction between B cells and CD4^+^ T cells (Cui et al., 2021). Maglioco et al. (2017), found that depleting B cells at different time points led to different outcomes for T cell responses and tumour growth in mice (Table 1). When B cells were depleted before tumour implantation, tumour growth increased due to more T_Regs_ within the TDLN (Maglioco et al., 2017). However, when B cells were depleted after tumour implantation, tumours regressed, and more cytotoxic CD8 T cells were found in the TDLN (Maglioco et al., 2017). In a mouse melanoma model, B cell depletion impaired T cell responses, which enhanced tumour growth (DiLillo et al., 2010). These studies elucidate that B cells cannot simply be lumped together because their functions can be spatiotemporally dependent and may vary among different types of tumours. The role of B cells in cancers has also been shown in patients. For instance, a study analysed TNBC samples (n = 269) to check tumour infiltrating B cells and plasma cells. They observed better disease-free survival in patients with above median level density of CD 38^+^ plasma cells (Yeong et al., 2018) (Table 1).

**TABLE 1:** B cells in cancer models.

Reference	B cell function in mouse cancer models	B cell phenotype	Cancer type	Prognosis
Ganti et al. (2015)	T2-MZP-like B_Regs_ promote tumour growth but the mechanism is not via IL-10 or T_Regs_	B220^+^ IgM^Hi^ CD21^Hi^ CD23^+^	Melanoma	Negative
Maglioco et al. (2017)	B cells function differs during cancer progression	CD20^+^ IL-10	Fibrosarcoma	Indeterminate
Yeong et al. (2018)	CD38^+^ plasma cells correlate with better DFS in TNBC patients	CD38^+^ CD138^+^	Breast cancer (TNBC)	Positive
Zhang et al. (2021)	B cells secrete GABA to promote TAMs which increased IL-10 and tumour growth	CD19^+^ CD20^+^ Some expressed CD138^+^ GABA	Colon cancer	Negative
Cui et al. (2021)	Antigen-specific B cells prime Tfhs to secrete IL-21 which promoted effector CD8^+^ T cell function	GC B cells	Lung cancer	Positive

T2-MZP (Transitional 2- marginal zone precursor); DFS (Disease free survival); TNBC (Triple negative breast cancer); GABA (Gamma-aminobutyric acid); TAMs (Tumour associated macrophages); Tfhs (T follicular helper cells); GC B (Germinal centre B cell).

B cells may also play unconventional roles in modulating responses in the tumours and TDLN. One study demonstrated that B cell-derived lymphotoxin enhanced castration-resistant prostate cancer growth by promulgating STAT3 signalling at regressing tumours Mauri and Ehrenstein, 2008). In a different study, Tacconi, 2021, showed that B cell depletion abrogated the protection conferred by CD169 macrophages in the TDLN (Tacconi et al., 2021), underscoring the indirect role of B cells in regulating tumour immunity. Lastly, a recent study found that B cells in the TDLN produced GABA, which polarised macrophages into IL-10^+^ tumour-associated macrophages (TAMs) (Zhang et al., 2021) (Table 1). In another study, it is theorised that in tumours with high B cell counts, and where antibody-dependent cellular cytotoxicity plays a role in combating tumour growth, cancer cells upregulate soluble MICA/B, which inhibit NK cells (Hu et al., 2019). Thus, B cells can modulate other types of cells to create an immunosuppressive microenvironment.

## 6 Immunoregulatory B cells in cancer

Immunosuppressive B cells were first described in the 1970s and are now broadly characterised as B_Regs_ (Mauri and Ehrenstein, 2008; Rosser and Mauri, 2015). Originally, B_Regs_ were described to produce IL-10 and, thus, were named B-10 cells. Later, CD5 became a defining marker of B_Regs_. Many studies attempted to use CD5 to characterise B_Regs_ because CD5 could promote B cell tolerance by negatively regulating BCR signalling (Bikah et al., 1996), CD5^+^ B cells can secrete IL-10 in an autocrine manner (Gary-Gouy et al., 2002), and CD5 expression was upregulated in chronic lymphocytic leukaemia (CLL), which correlated with immunosuppression. However, an emerging body of evidence suggests CD5^neg^ B cells could also perform regulatory functions. Since then, more markers have been used to identify B_Regs_, but their existence in humans remains elusive. Thus far, in addition to IL-10 being a defining feature for B_Regs_, B_Regs_ in humans may include transitional B cells (CD24^hi^ CD38^hi^) (Flores-Borja et al., 2013; Hasan et al., 2019) and ADO B cells (CD39^+^CD73^+^) (Kaku et al., 2014; Jeske et al., 2020). Functionally similar B_Regs_ have been reported in mice, but their surface markers differ. For instance, the mouse equivalent of CD24^hi^ CD38^hi^ B cells is B-10 cells (IgM^+^ CD1d^+^CD5^+/−^). Some newer B_Reg_ subsets with specific markers, such as IgM^+^ and IgA^+^ plasmablasts or plasma cells, have also been found in mice (Matsumoto et al., 2014; Shen et al., 2014; Shalapour et al., 2015; Lino et al., 2018). Like CD5^+^ B_Regs_, these subsets have also been documented to secrete IL-10 and are associated with negative prognosis in cancer patients (Table 2).

**TABLE 2:** B_Regs_ in cancer patients.

Reference	B_Reg_ function in cancer patients	B cell phenotype	Cancer type	Prognosis
Lindner et al. (2013)	Granzyme B-expressing B cells inhibit CD4 T cell proliferation	CD19^+^ CD38^+^ CD1d^+^ IgM^+^ CD147^+^ IL-10 Granzyme B Indoleamine 2,3-dioxygenase (IDO)	Breast cancer Cervical cancer Colorectal cancer Ovarian cancer Prostate cancer	Negative
Shalapour et al. (2015)	IgA^+^ plasma cells promote prostate cancer resistance to Oxaliplatin	CD138^+^ IgA^+^ IL-10 PD-L1^+^	Prostate cancer	Negative
Guan et al. (2015)	PD-L1 on cancer cells induces B_Regs_, which secrete IL-10 and promote T_Regs_	CD19^+^ CD24^+^ CD38^+^ IL-10	Invasive breast cancer	Negative
Guan et al. (2016)	PD-L1 is upregulated on B_Regs_ and correlates with increased T_Regs_	CD19^+^ CD24^+^ CD38^+^ IL-10 PD-L1^+^	Invasive breast cancer	Negative
Shalapour et al. (2017)	IgA^+^ plasma cells suppress CD8^+^ T cells	CD138^+^ IgA^+^ IL-10 PD-L1^+^ TGF-β	Liver cancer	Negative
Ye et al. (2018)	TIM-1^+^ B_Regs_ inhibit CD8 T cell IFNγ and TNFα production in co-cultures	CD5^hi^ CD24^−^ CD27^−/+^ CD38^+/hi^ IL-10 TIM-1^+^	Liver cancer Lung cancer	Negative
Chen et al. (2019)	B_Regs_ correlate with IL-10 to induce CD8^+^ T cell inhibition	CD19^+^ CD5^+^ CD1d^+^ IL-10	Cervical cancer	Negative
Gu et al. (2019)	αHSPA4 IgG promotes metastasis via the CXCR4/SDF1α axis	​	Breast cancer	Negative

Despite their elusive roles in cancer, several subsets of B_Regs_ have been shown to perform important roles in mouse models. A transitional B_Reg_ subset, immature transitional-2 (T2-MZP) B cells, were found to accumulate in the TDLN preferentially (Ganti et al., 2015). To determine whether T2-MZP B cells had a pro-tumoural function, Ganti et al. adoptively transferred various B cell subsets into B cell-deficient mice and found that T2-MZP B cells promoted melanoma tumour growth (Table 1). In prostate and liver cancer models, IgA^+^ plasma cells have been reported to express IL-10 and PD-L1, which suppress T cell response (Ganti et al., 2015; Shalapour et al., 2015; 2017). Although the existence of B_Regs_ in humans has been heavily contended, one recent study reported that B_Regs_ have been found in the TDLN of cancer patients. IL-10-producing B_Regs_ were found in the TDLN of oral squamous cell carcinoma patients. These IL-10^+^ B_Regs_ were higher in node-positive patients (Piersiala et al., 2022). Furthermore, B_Regs_ have been documented to convert CD4^+^ T cells to T_Regs_ independently of IL-10 (Olkhanud et al., 2011; Wang et al., 2015). Since B cells also function as APCs, tumour-infiltrating B_Regs_ may control T cell apoptosis or exhaustion through contact-mediated mechanisms such as the expression of PD-L1 (Guan et al., 2015; 2016; Zhang et al., 2016; Wang et al., 2019) (Table 2). However, insufficient distinct markers for B_Regs_ remain a limiting factor for studying B_Regs_ in the tumour and TDLN.

In summary, by understanding B_Reg_ subsets in the TDLN, their mechanisms, and their interactions with other immune cells, researchers can leverage this knowledge to target pathogenic B_Regs_ to ameliorate current ICI therapies. Additionally, more scrutiny is needed for excessive lymphadenectomy because the TDLN is an important therapeutic target, and patients may benefit from TDLN-targeted immunotherapies. Combining these aspects will allow scientists and physicians to fine-tune treatment regimens for patients.

## 7 Tertiary lymphoid structures (TLSs) serve as ectopic immunomodulatory sites in cancer

**FIGURE 5:**
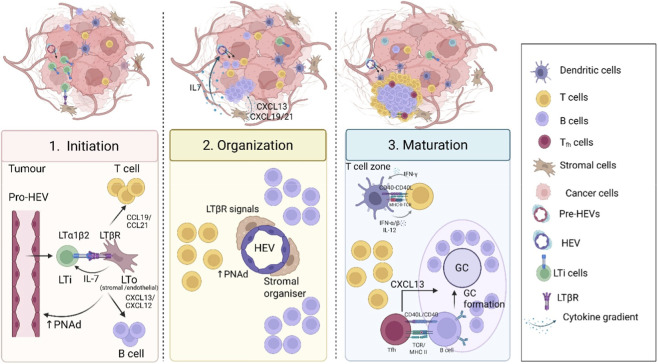
Steps in TLS formation: Briefly TLS formation in tumour takes place in three major steps: 1. Initiation: Pro-HEVs recruit LTi in response to inflammatory cytokines. LTi express LTα1β2 which interacts with LTβR on LTo. In return, LTo secretes IL-7 which activates the expression of adhesion molecules that helps in the recruitment of B and T cells. 2. Organisation: HEV and Stromal cells maturation in TLS, supporting immune cell homing and segregation in TLS through chemokine gradients. T cells follow CCL19/21 and B cells move towards CXCL 13/CXCL12 secreting cells. 3. Maturation: DCs activate T cells in the T cell zone, then some of the activated T cells start moving towards CXCL13 secreting cells by expressing CXCR5 these cells are known as T follicular cells (Tfh). Naïve B cells interact Tfh at the interfollicular zone and then undergo GC reaction. Created in https://BioRender.com.

Within tumours, B cells are often scarce however, when there is strong B cell presence, they often form aggregates in the tertiary lymphoid structures (TLSs). TLSs are ectopic lymphoid structures that resemble LNs and usually consist of B and T cell compartmentalisation and HEVs (Pitzalis et al., 2014; Montorsi et al., 2022). Additionally, B cell-derived lymphotoxin alpha and beta complex (LTα_1_β_2_) can engage lymphotoxin β receptor (LTβR) expressed by stromal cells to induce tertiary lymphoid structures (TLSs) formation (McLaughlin et al., 1998; Mellman, 2013) Their main cellular components include CD20^+^ B cells, follicular dendritic cells (FDC), fibroblasts, high endothelial venules (HEV), and CD4^+^ and CD8^+^ T cells (Figure 5). In tumours, TLS serves as a local site for antigen presentation, facilitating the differentiation of T and B cells into effector and memory cells, thereby transforming an immune-cold tumour environment into an immune-hot one (Sautès-Fridman et al., 2019). Recently, there has been a resurgence of interest in B cells in cancer because TLS in cancer is associated with favourable prognosis in patients in multiple cancers. TLS can be classified as immature, which lack complexity, or mature often comprising of FDCs, T_fh_, forming GC-like structures and can sometimes include lymphoid organ-specific structures such as HEVs. Mature TLSs in cancer frequently exhibit LN-like functions such as immune cell activation.

Heterogeneity of TLSs is a broad yet promising area for improving cancer prognosis. In tumours, TLS number, density, location, and maturation can vary according to tumour stage and tumour type (Lauss et al., 2022). In patients, B cells in mature TLSs are locally activated, forming GCs and differentiation into memory B cells and plasma cells, which are associated with better ICI responsiveness (Cabrita et al., 2020; Helmink et al., 2020; Petitprez et al., 2020). A study visualised lymphoid aggregates in whole tumour sections of previously untreated high-grade serous ovarian cancer (n = 30). They assessed colocalization patterns of CD8^+^ tumour infiltrating lymphocytes (TILs) to characterised TLS into 4 different types of lymphoid aggregates. Type1 aggregates detected CD4 and CD8 T cells, CD20 B cells and CD208 DCs (n = 17/30). Type 2 aggregates do not contain DCs and B cells formed diffused patterns with no clear follicle and clear T cell zone (n = 17/30). Type 3 aggregates contain mature TLS with GCs with CD21 FDCs and outer T cell zone(n = 7/30). Type 4 aggregates contain GCs without clear T cell zone (n = 7/30). Type III tumours showed 60% diseases specific survival and no TIL tumours 10%–20% (Kroeger et al., 2016). On the contrary, a hepatocellular carcinoma study analysed a cohort of 82 patient biopsies and identified that TLS depends on NF-κB signalling. Long term IKK activation in hepatocytes form TLS and aggressive HCC (Finkin et al., 2015) (Table 3).

**TABLE 3:** Tertiary lymphoid structures (TLS) and cancer patient prognoses.

Reference	B cell subsets	TLS definition	Clinical information	Prognosis
Finkin et al. (2015)	B cells	TLS with organised clusters of B cells, T cells and HEVs	Hepatocellular carcinoma n = 82	Negative
Kroeger et al. (2016)	TIL-Bs TIL-PCs	TLS were clustered into 4 subgroups: I: Small clusters of B cells, T cells and DCs II: Large clusters of B cells and T cells without follicles or distinct zones III: TLS with organised clusters of B cells, T cells and GC-like structures IV: TLS with organised clusters of B cells and GC-like structures but lack T cells	Ovarian cancer n = 172 and dataset from the TCGA, n = 570	Positive
Garaud et al. (2019)	TIL-Bs Memory B cells Plasma cells IgG B cells GC B cells	TLS with organised clusters of B cells, T cells and DCs	Breast cancer n = 117	Positive
Noël et al. (2021)	Memory B cells Naïve B cells Plasma cells GC B cells Tfh	TLS with organised clusters of B cells, T cells and GC-like structure	Breast cancer n = 50	Positive

Many immune cells have functional plasticity; hence, B cells may also exhibit divergent phenotypes as a result of their microenvironments. B cells have been theorised to be critical for TLS formation because they are a crucial source for LT signals, and more studies on how B cells and TLS impact therapeutic outcomes should be investigated. By understanding how B cells may be altered during cancer progression as well as pre- and post-therapy, we may be able to improve our current immunotherapies or identify patient cohorts that are better suited for specific types of therapies.

### 7.1 Formation of TLS

Typically, TLS formation involves communication between lymphoid tissue initiator (LTi) and lymphoid tissue organizer (LTo) cells. TLS formation generally undergoes three main development phases: initiation, structural organisation and functional maturation (Deng et al., 2025). In phase I, high endothelial venules (HEV) recruit lymphocytes, LTi and DCs in response to inflammatory cytokines (IL-1β, IL-6, and TNF-α). Under different conditions, these LTi and LTo cells can be different cell types (Deng et al., 2025; Li et al., 2025). LTi cell-derived lymphotoxin alpha and beta complex (LTα1β2) can engage lymphotoxin β receptor (LTβR), expressed by stromal cells and endothelial cell (Lochner et al., 2011) Figure 5.

Stromal cells secrete IL-7 that activates LTαβ2 secretion from lymphocytes, which binds to LTβR on LTo and induces secretion of adhesion molecules such as peripheral node addressin (PNAd) on HEV. Stromal cells express VCAM-1 and ICAM-1, along with chemokines like CXCL13, CXCL12 and CCL19/21. CXCL13 and CCL21 recruit B cells and T cells by binding to CXCR5 and CCR7, respectively (Khanal et al., 2023; Zhao et al., 2024). In melanoma, Rodriguez et al. reported that FAP^−^ VCAM^+^ APRIL^+^ CAF (Cancer-Associated Fibroblasts) cells act as LTo when implanted intraperitoneally (i.p) in mice, and LTαβ2 expressed by B cells mediates their recruitment to TLS. CAF in tumours developed via i.p. route secrete more CXCL13 than those implanted via subcutaneously and recruits more B cells. Their results conclude that ICI therapy is correlated with reduction in the tumour size of I.P. tumours and increase in TLS number. They also worked out that TNFR^−/−^ mice lack TLS, suggesting the important role of TNFR signalling in TLS synthesis (Rodriguez et al., 2021). Another study on atherosclerosis reported that M1 macrophages act as LTi and induce vascular smooth muscle cells to act as organizer cells through TNFR signalling, rather than the classical lymphotoxin axis (Rodriguez et al., 2021). However, whether cancer-associated macrophages can function as LTi remains unknown.

The second phase involves spatial segregation of TLS cells (Deng et al., 2025) Lymphocyte recruitment requires a gateway, which is formed by the synthesis of HEVs from endothelial cells. As illustrated in Figure 5 synthesis of HEVs is a collaboration between stromal cells and lymphocytes, where lymphocytes activate them by promoting TNFR signalling and LIGHT secretion. Stromal cells activate ECs in a paracrine manner through IL-7Ra (Zhao et al., 2024) Studies validated three categories of TLS based on three markers: CD20 (naïve B cell marker), CD21 (immature FDC) and CD23 (mature FDC). So, TLS is categorised as CD20^+^ TLS lymphoid aggregates, CD20^+^CD21^+^ non-GC TLS and CD20^+^CD21^+^CD23^+^ GC (mature) TLS (Vanhersecke et al., 2021; Werner et al., 2021; Le Rochais et al., 2023). Although in different GC maturation states, both CD21^+^ FDC and CD21^+^ CD23^+^ FDC present antigen to B cells, the former promotes low B cell maturation and low efficacy antibodies (Fridman et al., 2022). A study on a prostate cancer population used spatial transcriptomics to detect TLS heterogeneity. They described the mature TLS phenotype as having good architecture, CD20^+^ B cells in the inner region surrounded by T cells, and the presence of FDC and GC cells. Notably, mature TLS had a lower incidence of colocalization between B cells and T_reg_ cells than immature TLS. In fact, colocalization of tumour-associated macrophages (TAMs) and CAFs with B cells is higher in immature TLS, creating an immunosuppressive milieu. Also, intratumoral TLS (intra-TLS) is more developed than peripheral TLS and peri-TLS has a greater incidence of Tp53 and FOXA1 mutations, suggesting that anti-tumour strength is determined by intra-TLS GCs (Wang et al., 2025). Mature FDC are highly efficient in trapping and presenting antigen to B cell by Fc receptor and better at promoting GC B cell survival, proliferation and differentiation through secretion of differentiation signals like BAFF, APRIL and IL-6. However, precisely how FDC matures in TLS is still under research but, activated local stromal cells are observed to differentiate into FDC after coming in contact with immune cells in TLS (Bao et al., 2024).

In the third phase, CD4^+^ T cells differentiate into T_fh_ cells. Stromal cells produce CXCL13 which recruits CXCR5^+^ PD1^+^ ICOS^+^ pre-T_fh_ cells. Next, classical DC1 presents antigen to CD4^+^ T cells within TLS to facilitate T_fh_ differentiation via TGF-β- SATB1 and IL-6/IL-12 signalling (Sautès-Fridman et al., 2019; Chaurio et al., 2022; Deng et al., 2025). However, the exact mechanistic signalling that drives T_fh_ differentiation in TLS is not well defined (Mattiuz et al., 2024). Similar to SLOs CD40^−^CD40L-based interaction between T_fh_ and B cells is crucial for GC formation and production of high-affinity antibodies Figure 5 (Bao et al., 2024). IL-21, secreted by T_fh_ in TLS, also helps B cells in GC reactions (Luo et al., 2024). Inside TLS, CCL19^+^ FRCs create an intratumoral T cell environment crucial for antitumour immunity, form an interconnected network in TLS and T cell tracks (Acton et al., 2021; Onder et al., 2025). Clearly, T_fh_ plays an important role in TLS and anti-tumour immunity, so more reliable GC-competent T_fh_ biomarkers need to be identified to distinguish Tfh from bystander clusters (Song and Craft, 2023). Based on tumour histologies (n = 93), a sarcoma study concluded that GC is a feature of secondary TLS, and that earlier stages are named as primary TLS and early TLS, with 60.5% early, 21.1% primary, and 18.3% secondary TLS (Petitprez et al., 2020). This categorisation was also used for colorectal cancer and urothelial cancers (Lauss et al., 2022). In another melanoma study, it was validated that both GC-negative TLS (immature) and GC-positive TLS (mature) can coexist within the same tumour (Cabrita et al., 2020).

### 7.2 B-cell heterogeneity in TLS

B cells are active participants in shaping tumour immunity; they can either promote or hinder tumour progression, depending on factors such as antibody isotype, B cell subset, and the tumour environment. In the tumour microenvironment (TME), certain B-cell subtypes require activation, such as naïve B cells (CD19^+^ CD20^+^ IgD^+^), while B_Regs_ (CD19^+^ CD20^+^ IL10^+^ CD11b^+^CD1d^hi^ CD5^+^ TIM-1^+^) require suppression. In addition to these two cell types, memory B cells (CD19^+^ CD20^+^ CD27^+^ IgM^+^), switched memory cells (CD19^+^ CD20^+^ CD27^+^ IgA/G^+^), GC B cells (CD19^+^ CD20^+^ BCL6^+^ CD95^+^ CD38^neg^ GL7^+^), plasmablasts (CD19^+^ CD20^+^ CD27^+^ CD44^hi^ CD38^+^ CD138^+^ Ki67^+^) and plasma cells (CD19^+^ CD38^+^ CD138^hi^ CXCR3^+^ BCMA^+^ MUM1^+^) are the most commonly found B cell subtypes in TME (Dang et al., 2022; Fridman et al., 2022). A detailed single-cell sequencing of tumour infiltrating B cells (TIL-Bs) (cells 8774) from breast cancer tissues grouped B cells into five clusters based on their marker expression, with naïve B cell-like markers (MS4A1, TCL1A, BCL6, LMO2^hi^, CD22, CR2), GC B cell-like markers (MK167^hi^, HMGB2^hi^ and STMN1^hi^), CXCR4^+^ B cells (CXCR4^hi^, BANK1^hi^), follicular B cells (NR4A1/2, CD69), and Plasma B cells (PRDM1, XBP1, MZB1, IGHM, CD27 and SSR4). This study suggested that upregulated CD69, a follicular B cell marker, correlated with better overall survival, as verified with TCGA data (Wang et al., 2023). A comprehensive B-cell investigation in head and neck squamous cell carcinoma (HNSCC) analysed TILs from HPV^+^ and HPV^−^ HNSCC and found that SEMA4A^+^ GC TILs, as well as the number of GC in TLS, are increased in HPV^+^ HNSCC compared to HPV^−^ HNSCC. These findings indicated that SEMA4a, a membrane glycoprotein, forms immune aggregates by interacting with endothelial cells and by stimulating T cells (Ruffin et al., 2021). These findings suggest that identifying TIL-Bs could help us improve patient outcomes by harnessing precise medicine.

### 7.3 Therapeutic importance of B cells in TLS

Previous studies have shown that tumours containing high tumour infiltrating lymphocytes (immune-hot) often contain TLS and are associated with improved overall patient survival, particularly when combined with ICI therapies (Petitprez et al., 2020). This sarcoma study has classified TME into 5 different categories based on its composition and labelled them as A, B, C, D and E. A and B are immune-low, D and E are immune high, and C is a highly vascularised sarcoma group, where patients with sarcoma immune cell (SIC) A had poorer survival than SIC D and E. Additionally, they have also separated tumours based on elevated and downregulated cytotoxic lymphocytes, CD8^+^ T cells, and B lineage markers, indicating that high B cell levels are associated with improved survival, regardless of CD8^+^ T cell levels (Petitprez et al., 2020). Table 4 highlights the prognostic relevance of TLS in cancer patients treated with immune checkpoint inhibitors (ICIs). However, the mechanism by which TLSs regulate ICI therapy remains unclear.

**TABLE 4:** Prognostic value of TLS in cancer patients treated with immune checkpoint inhibitors (ICIs).

Reference	B cell subsets	TLS definition	Immunotherapy	Clinical information	Prognosis
Cottrell et al. (2018)	Plasma cells	TLS with organised clusters of B cells, T cells and GC-like structure	Neoadjuvant ICI anti-PD-1	NSCLC n = 20	Positive
Helmink et al. (2020)	Memory B cells Naïve B cells Plasma cells class-switched memory B cells	TLS include B cells (CD19/20+), T cells (CD3^+^), FDCs (CD21^+^) and HEVs	ICIs including anti-PD-1, anti-CTLA4 and combined therapies	Metastatic melanoma n = 23 • Nivolumab n = 12 • Nivolumab and Ipilimumab n = 11 Metastatic RCC n = 104 • Randomised drug trial	Positive
Cabrita et al. (2020)	Memory B cells Naïve B cells Plasma cells GC B cells	TLS with organised clusters of B cells, T cells and GC-like structures	ICIs including anti-PD-1, anti-CTLA4 and combined therapies	Melanoma n = 177 • Regional metastasis: n = 104 Distant metastasis: n = 50 • *In situ*: n = 19 • Unknown: n = 4	Positive
Petitprez et al. (2020)	​	TLS with organised clusters of B cells, T cells and GC-like structures	ICI anti-PD-1	Soft-tissue sarcoma biopsies n = 47 Extended studies, n = 869	Positive
Budczies et al. (2021)	B cells	TLS B cells (CD20^+^) and gene expression of CD19	ICI anti-PD-1, anti-PD-L1 and combined therapies	Metastatic lung adenocarcinoma n = 43	Positive
Vanhersecke et al. (2021)	B cells	TLS with organised clusters of B cells, T cells and GC-like structures are classified as mature TLS are classified as immature if they lack FDCs (CD23^+^)	ICI anti-PD-1, anti-PD-L1 and combined therapies	Pan-cancer n = 328 NSCLC • TLS: n = 37 • No TLS: n = 90 Soft-tissue sarcoma • TLS: n = 8 • No TLS: n = 38 Bladder cancer • TLS: n = 15 • No TLS: n = 16 Colorectal cancer • TLS: n = 11 • No TLS: n = 16 Head and neck carcinoma • TLS: n = 5 • No TLS: n = 7 Renal carcinoma • TLS: n = 2 • No TLS: n = 8 Breast carcinoma • TLS: n = 1 • No TLS: n = 6 Others • TLS: n = 26 • No TLS: n = 42	Positive

TLS (Tertiary lymphoid structure); GC (Germinal centre); FDC (Follicular Dendritic Cells); HEV (High endothelial venules); ICI (Immune checkpoint inhibitor); NSCLC (Non-small cell lung cancer).

Moreover, A plasma cell cohort analysis in clear cell RCC primary tumours (n = 130) from three separate ICI therapy groups with Nivolumab (Anti-PD-1) and Ipilimumab (anti-CTLA4) showed that plasma cells develop from B cells inside the TLS GC region of malignancies. They have also shown that murine plasma cells have low CXCR5; therefore, instead of CXCL13-dependent dissemination from TLS, they follow the CXCL12^+^ fibroblastic track. They also discovered a favourable connection between IgG-labelled tumour cells and response to ICI, along with progression-free survival (PFS) in patients receiving the combination of Nivolumab and Ipilimumab, or Nivolumab alone (Meylan et al., 2022). Similarly, a hepatocellular carcinoma study (HCC) reported an association between TLS and neoadjuvant ICI-treated HCC. They identified CD20^+^CXCL13^+^ lymphocyte aggregates as potential predictors of response to immunotherapy and an alternative for TLS detection. Moreover, they also inferred that the convoluted TLS phenotype, characterised by dispersed B cells and compact T cells, is due to the expansion of tissue-resident CD8^+^ T cells and disruption of the T cell zone in the late stage of TLS, which may support the memory phase of adaptive tumour response (Shu et al., 2024). Immune cells from TLS-positive and TLS-negative tissues were examined in a scRNA seq study of breast cancer, which found CD23 to be a mature TLS marker associated with FDC. The findings indicated that although TLS-negative groups are associated with immunosuppressive environments (characterized by the upregulation of TGF-β, CD86, CD80, BAFF, CTLA4, and CD137), TLS-positive groups are linked to more favourable outcomes. They provided evidence in favour of the notion that TLS exhibits a better prognosis than TIL-B cells (Wang et al., 2023). A better prognosis is connected not only with a higher number of TLS, but also with the geographical location of TLS in the TME, which influences patient response to ICIs. According to an HGSOC study, cells that are often resistant to ICI contain more terminal mature TLS (mTLS) than stroma. They also compared early TLS (eTLS) and mTLS in HGSOC to those in NSCLC and discovered that they are less common in HGSOC, making them more vulnerable to ICIs. The ICI-resistant HGSOC environment contains more TIM3^+^PD1^+^CD8^+^ T cells than TCF1^+^PD1^+^CD8^+^ T cells, and TLS is undeveloped, indicating a lack of B cell and T cell activation in TME. Furthermore, they observed that higher tumour burden was connected with TLS production in both human HGSOC and mouse ovarian cancer models, CD 20^+^ B cells, CD8^+^ T cells, and TLS-associated chemokine genes, indicating more ICI responsiveness (Kasikova et al., 2024).

Currently, a wide array of immunotherapies with demonstrated efficacy in cancer treatment are available (Table 5). In this review, we have addressed the heterogeneity of the TME, underscoring that a uniform sequence and therapeutic approach for all cancer patients is inherently flawed. TIL-B have been shown to play a pivotal role within the TME in modulating tumour progression. However, our understanding of TIL-B biology and function remains limited and further investigation is essential to develop targeted therapeutic strategies that can improve clinical outcomes.

**TABLE 5:** Summary highlighting different types of cancer immunotherapy and proven efficacy in cancer.

Immunotherapy	Example	FDA	Type of cancer	Dose and route of administration	Adverse effects	References
Cancer vaccine	BCG vaccine	No	Bladder cancer	-	-	Redelman-Sidi et al. (2014), Green et al. (2018)
	Sipuleucel-T (DC-loaded vaccine against prostatic acid phosphatase)	Yes	Prostate cancer	I.V. 3 doses at 2-week intervals One dose contains 50 million autologous CD54 + cells activated with PAP-GM-CSF, suspended in 250 mL of Lactated Ringer’s injection	Chills, fatigue, fever, back pain, nausea, joint ache and headache	Kantoff et al. (2010)
Cytokine therapy	IL-2	Yes	Melanoma Renal cancer	600,000IU/kg (0.037 mg/kg) every 8 h by 15 min IV	Fever, chills, nausea, vomiting, pruritus, rash, diarrhoea, hypotension, oedema and oliguria	Rosenberg (2014)
Oncolytic viral therapy	T-VEC (Talimogene laherparepvec) (Modified herpès simplex virus T1)	Yes	Melanoma	starting concentration: 1 million PFU per ml (4 mL) subcutaneous or nodal lesion injection	Fatigue, chills, pyrexia, nausea, influenza-like illness and injection site pain	Johnson et al. (2015)
Monoclonal antibody	Rituximab (αCD20)	Yes	Leukaemia Lymphoma	375 mg/m^2^ (Non-Hodgkin Lymphoma, Chronic Lymphocytic leukemia) IV	Fatal infusion reactions include (Mucocutaneous reactions, HPV reactivation and progressive multifocal leukoencephalopathy), neutropenia, lymphopenia and asthenia	McLaughlin et al. (1998), Maury et al. (2016)
	Trastuzumab (αHER-2)	Yes	Breast cancer, metastatic gastric cancer	breast cancer- initial dose: IV 4 mg/kg over 90 min then 2 mg/kg over 30 min weekly for 52 weeks Metastatic Gastic cancer: initial dose of 8 mg/kg over 90 min followed by 6 mg/kg over 30–90 min every 3 weeks	Infusion reaction (cardiomyopathy, infusion reaction and pulmonary toxicity)	Piccart-Gebhart et al. (2005)
	Bevacizumab (αVEGF)	Yes	Metastatic colorectal cancer Non-squamous non-small cell lung cancer, metastatic breast cancer	MCC: 5 mg/kg IV every 2 weeks with bolus- IFL, 10 mg/kg with FOLFOX4 NSCLS: 15 mg/kg every 3 weeks with paclitaxel/carboplatin Metastatic BC: 10 mg/kg IV every 2 weeks with paclitaxel Glioblastoma: 10 mg/kg IV every 2 weeks	Epistaxis, rhinitis, proteinuria, rectal haemorrhage, lacrimation disorder	Glade Bender et al. (2008)
Checkpoint inhibitor	Ipilimumab (αCTLA-4)	Yes	Melanoma Renal cancer Colorectal cancer Hepatocellular carcinoma NSCLC	Metastatic melanoma: 3 mg/kg every 3 weeks (4 doses) RCC: 1 mg/kg + nivolumab 3 mg/kg HCC, NSCLC: 3 mg/kg + Nivolumab 1 mg/kg	Immune-mediated adverse reactions, enterocolitis, hepatitis, dermatitis (including toxic epidermal necrolysis), neuropathy, and endocrinopathy	Hodi et al. (2010), Boutros et al. (2016)
	Pembrolizumab (αPD-1)	Yes	Melanoma Renal cancer Bladder cancer Colorectal cancer Hodgkin lymphoma NSCLC	200 mg every 3 weeks	Musculoskeletal pain, pyrexia, pruritus, dyspnea	Brahmer et al. (2012), 2015; Motzer et al. (2015), Boutros et al. (2016), Le et al. (2017)
	Nivolumab(αPD-L1)	Yes	Melanoma, NSCLC	Melanoma: 240 mg every 2 weeks NSCLC: 360 mg every 3 weeks	Immune-mediated adverse reactions, embryo-fetal toxicity, complications of allogeneic, and infusion-related reactions	Boutros et al. (2016), Brahmer et al. (2015), Le et al. (2017), Motzer et al. (2015)
	Atezolizumab (αPD-1)	Yes	Urothelial carcinoma, NSCLC, HCC	UC, NSCLC, HCC: 840 mg every 2 weeks NSCLC	Immune-mediated adverse reactions, embryo-fetal toxicity, complications of allogeneic, and infusion-related reactions	Boutros et al. (2016), Brahmer et al. (2015), Le et al. (2017)

NSCLC (non-small cell lung cancer); HCC (Hepatocellular carcinoma); RCC (Renal cell carcinoma); UC (Urothelial carcinoma); MCC (Merkel cell carcinoma).

## 8 Concluding remarks

Much of the cancer research focuses on the tumour or primary site. Therapeutic interventions discussed in Table 5 have also focused on reactivating immune cells in the tumours. Nevertheless, the tumour vasculature is disorganised, which may result in low drug penetration. The desired anti-tumour effect will be limited if the drug cannot effectively reach tumour-infiltrating immune cells. The TDLN is an essential site for anti-tumour responses, but accumulating data show immunosuppressive T cells can also be primed and expanded in the TDLN. Recent studies have slowly unravelled how antigen-specific T cells are primed in the TDLN before trafficking to the tumour to elicit responses. Meanwhile, administering ICI therapies to sites that will inherently drain to the TDLN increases the likelihood of reactivating tumour-specific immune cells, particularly if it is coupled with irradiation. Evidence in mice suggests that targeting the TDLN with ICIs achieves a similar efficacy as systemic delivery whilst reducing drug toxicity. Should ICI therapy targeting sentinel lymph nodes become a new standard for all cancer patients, or what biomarkers can reliably determine which patients and/or at which disease stages would benefit the most from this novel approach?

Although targeting the TDLN has been shown to be beneficial in animal studies, the underlying mechanism remains unclear. Dissecting the mechanisms of immunosuppression in the TDLN and unravelling the complex cell interactions, such as those between antigen-presenting cell subsets, T cells, and stromal cells within the TDLN would reveal how to selectively interrupt immunosuppressive circuit. This may ultimately enable the discovery and develop new therapies that extend beyond current ICI-based therapies. Furthermore, while patients with solid tumors have sentinel LNs, TLSs develop *de novo* within tumours in only a subset of patients. Despite strong evidence shows that TLSs are associated with favourable prognosis and positive responses to ICI immunotherapy, it remains clear how insights gained from TDLN can be leveraged to induce or reprogram TLSs. A key question is whether engineering TLSs could enable precise, site-specific immune modulation, thereby transforming them from predictive indicators into useful therapeutic targets. Future strategies may integrate nanotechnology driven drug delivery methods that locally boost immune activation to improve the therapeutic index, alongside liquid biopsies and molecular imaging to monitor *in vivo* alterations in TLS/TDLN.
